# BNT162b2 against COVID-19 in Brazil using a test-negative design: Study protocol and statistical analysis plan

**DOI:** 10.1371/journal.pone.0276384

**Published:** 2022-10-20

**Authors:** Regis Goulart Rosa, Julia Spinardi, Kristen E. Allen, Josélia Manfio, Cintia Laura Pereira de Araujo, Mírian Cohen, Caroline Cabral Robinson, Daniel Sganzerla, Diogo Ferreira, Emanuel Maltempi de Souza, Jaqueline Carvalho de Oliveira, Daniela Fiori Gradia, Ana Paula Carneiro Brandalize, Gabriela Almeida Kucharski, Fernando Pedrotti, Cristina de Oliveira Rodrigues, Moe H. Kyaw, Graciela del Carmen Morales Castillo, Amit Srivastava, John M. McLaughlin, Maicon Falavigna

**Affiliations:** 1 Social Responsibility Unit, Hospital Moinhos de Vento (HMV), Porto Alegre, RS, Brazil; 2 Research Unit, Inova Medical, Porto Alegre, RS, Brazil; 3 Pfizer, Vaccines Medical and Scientific Affairs–Emerging Markets, Collegeville, PA, United States of America; 4 Research Institute, HMV, Porto Alegre, RS, Brazil; 5 Unimed, Porto Alegre, RS, Brazil; 6 Otus Solutions, Porto Alegre, RS, Brazil; 7 Universidade Federal do Paraná (UFPR), Curitiba, PR, Brazil; 8 Secretaria Municipal de Saúde de Toledo, Toledo, PR, Brazil; 9 Department of Health Research Methods, Evidence, and Impact, McMaster University, Hamilton, Ontario, Canada; Beth Israel Deaconess Medical Center, UNITED STATES

## Abstract

**Introduction:**

Real-world data on COVID-19 vaccine effectiveness are needed to validate evidence from randomized clinical trials. Accordingly, this study aims to evaluate, in a real-world setting in Brazil, the effectiveness of Pfizer-BioNTech BNT162b2 against symptomatic COVID-19 and COVID-19-related complications across diverse populations.

**Materials and methods:**

A test-negative case-control study with follow-up of cases is currently being conducted in Toledo, a city in southern Brazil, following a mass COVID-19 vaccination campaign with BNT162b2. The study is being conducted among patients aged 12 years or older seeking care in the public health system with acute respiratory symptoms and tested for SARS-CoV-2 on reverse transcription polymerase chain reaction (RT-PCR). Cases are RT-PCR positive and controls RT-PCR negative. Test-positive cases are prospectively followed through structured telephone interviews performed at 15 days post-enrollment, and at 1, 3, 6, 9 and 12 months. Baseline demographic, clinical, and vaccination data are being collected by means of structured interviews and medical registry records reviews at the time of enrollment. All RT-PCR-positive samples are screened for mutations to identify SARS-CoV-2 variants.

**Ethics and dissemination:**

The study protocol has been approved by the research ethics committee of all participant sites. Study findings will be disseminated through peer-reviewed publications and conference presentations.

**Trail registration:**

**Clinicatrials.gov:** NCT05052307.

## Introduction

COVID-19, caused by SARS-CoV-2 infection, has affected millions of people around the world. In Brazil, the number of cases has surpassed 28 million, with more than 640,000 deaths from COVID-19 by February 2022 [1]. Therefore, mass-vaccination campaigns using approved COVID-19 vaccines have become a health priority [2].

The Pfizer-BioNTech mRNA COVID-19 vaccine BNT162b2 was approved on June 11, 2021 by the Brazilian National Health Surveillance Agency (ANVISA) for persons 12 years of age and older [3]. A randomized, controlled trial involving 43,448 participants aged 16 years or older showed that a two-dose 21-days apart regimen of BNT162b2 demonstrated 95% efficacy (95% confidence interval [CI], 90% to 98%) against laboratory-confirmed SARS-CoV-2 infection [4]. Related adverse events were reported by 21% of participants (mainly pain at the injection site, fatigue and headache), and were mostly mild-to-moderate in severity. A subsequent randomized, controlled trial involving 2,260 adolescents aged 12 to 16 years showed that a two-dose 21-days apart regimen of the BNT162b2 vaccine demonstrated 100% efficacy (95% CI, 75% to 100%) against laboratory-confirmed SARS-CoV-2 infection, with a safety profile characterized mainly by mild-to-moderate injection-site pain, fatigue and headache [5]. However, evidence regarding effectiveness of BNT162b2 in Brazil, especially against new variants of concern such as Omicron (currently the most prevalent lineage) [6] is limited; likewise, the long-term protection conferred by BNT162b2 vaccination and impact on COVID-19 severity and prolonged symptoms are not yet available.

## Objectives

### Primary objective

The primary aim of this study is to assess the effectiveness of BNT162b2 in preventing symptomatic COVID-19 in a real-world setting in Brazil.

### Secondary objectives

The secondary objectives are to assess the impact of BNT162b2 on: (1) symptomatic SARS-CoV-2 infection caused by circulating variants (e.g., Delta, and Omicron); (2) duration of COVID-19 symptoms; (3) hospitalization for COVID-19; (4) need for ICU admission; (5) need for invasive mechanical ventilation; (6) death from COVID-19; (7) health-related quality of life; (8) persistent COVID-19 symptoms; and (9) symptomatic SARS-CoV-2 reinfection.

## Materials and methods

This study protocol was registered at Clinicaltrials.gov before the enrollment of the first participant (NCT05052307).

### Study setting

This study has been conducted in Toledo (24° 42′ 50″ S, 53° 44′ 34″ W), a city in the state of Paraná, southern Brazil. The population of Toledo was estimated at 144,601 people in 2021. As of 05 February 2022, COVID-19 has affected 38,614 people with 478 deaths. The city secretary of health initiated an age-based COVID-19 vaccination campaign on 20 January 2021, starting with adults 16 years of age and older. A mass vaccination campaign using BNT162b2 for all nonimmunized individuals aged ≥ 12 years was initiated on 27 August 2021 using a two-dose schedule administered 21 days apart. A third dose (i.e., booster dose) of BNT162b2 has been recommended for healthy (according to an age-based prioritization strategy) and immunocompromised individuals aged ≥ 12 years since 15 September 2021. Other available COVID-19 vaccines in Toledo include CoronaVac (Sinovac), ChAdOx1 nCoV-19 (AstraZeneca), and Ad26.COV2.S (Janssen); around 50% of the vaccine-eligible individuals immunized against COVID-19 have received BNT162b2.

Toledo was chosen due to the city standard of care, which provides free healthcare access to a wide variety of COVID-19 diagnosis and management services, and due to the feasibility of accessing information on people who were tested for SARS-CoV-2 through the city surveillance system.

### Study design

**Fig 1:**
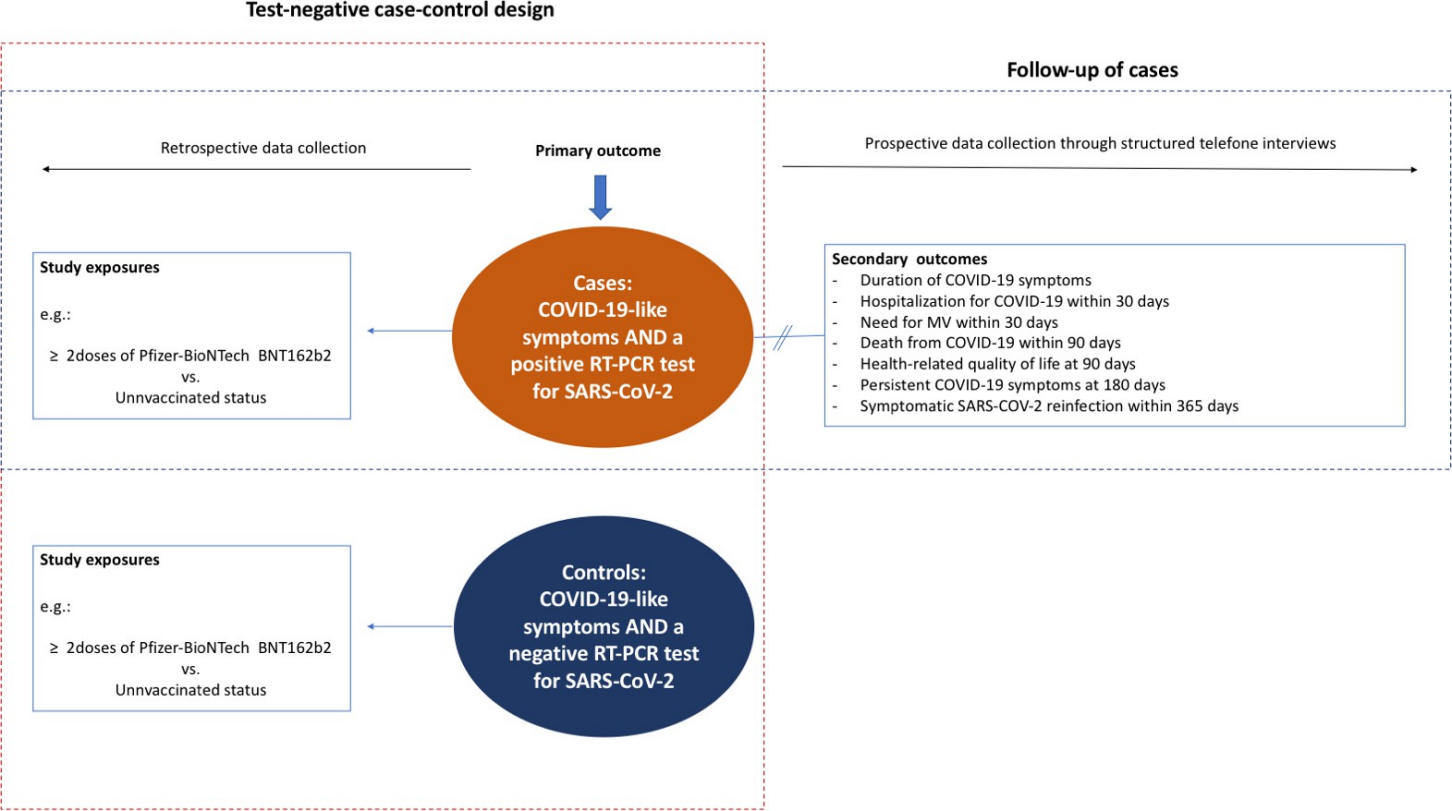
Study design. MV, mechanical ventilation; RT-PCR, reverse transcriptase polymerase chain reaction; SARS-CoV-2, severe acute respiratory syndrome coronavirus 2.

The present study is a test-negative case-control study with follow-up of cases (Fig 1). In this design, the same clinical case definition is used for both cases (i.e., participants with symptoms consistent with COVID-19) and controls (i.e., participants without the outcome) [11]. Reverse transcription polymerase chain reaction (RT-PCR) test for SARS-CoV-2 is prospectively used to distinguish cases from controls. Vaccination status and potential confounders are retrospectively ascertained among cases and controls through structured interviews and medical records review. Test-positive cases are prospectively followed through structured telephone interviews performed at 15 days, and at 1, 3, 6, 9 and 12 months.

### Participants

The present study population is composed of consecutive individuals aged 12 years or older who seek the public healthcare system of Toledo with symptoms suggestive of COVID-19.

Inclusion criteria are (1) age ≥ 12 years old; (2) to be a resident of Toledo; (3) seeking care in the public healthcare system of Toledo with symptoms suggestive of COVID-19 defined as follows: acute respiratory infection symptoms (nasal congestion, rhinorrhea, anosmia, sore throat, hoarseness, new or increased-from-baseline cough, sputum production, dyspnea, wheezing, myalgia) OR admitting diagnosis suggestive of acute respiratory infection symptoms (pneumonia, upper respiratory infection, bronchitis, influenza, cough, asthma, viral respiratory illness, respiratory distress, respiratory failure), and (4) having a nasopharyngeal (NP) or nasal sample for SARS-CoV-2 diagnosis obtained as standard of care.

Patients with history of remdesivir treatment in the past 30 days, convalescent plasma treatment in the past 90 days, treatment with monoclonal antibodies for COVID-19 in the past 90 days (e.g., sotrovimab, casirivimab-imdevimab, bamlanivimab, etesivumab), and those who refused to provide informed consent are excluded. Participants are allowed to re-screen; however, the follow-up is performed considering only the first enrollment of each individual for the study.

### Exposures

The primary exposure is the receipt of two or more doses of BNT162b2 according to the Brazilian National Immunization Program (BNIP) recommendations (with the receipt of the second dose 7 days or more before the onset of symptoms of COVID-19-like illness).

Secondary exposures are as follows: (1) receipt of one or more doses of BNT162b2 (with the receipt of the first dose 14 days or more before the onset of COVID-19-like symptoms); (2) receipt of one dose of BNT162b2 (with the receipt of dose, 14 days or more before the onset of COVID-19-like symptoms); (3) receipt of two doses of BNT162b2 (with the receipt of the second dose 7 days or more before the onset of COVID-19-like symptoms); (4) receipt of three doses of BNT162b2 (with the receipt of the third dose 7 days or more before the onset of COVID-19-like symptoms); (5) complete vaccination with other available COVID-19 vaccines in Toledo (CoronaVac [Sinovac], ChAdOx1 nCoV-19 [AstraZeneca], and Ad26.COV2.S [Janssen]) according to Brazil National Immunization Program (BNIP) recommendations.

The reference exposure group is composed of participants who had not received a first vaccine dose before the date of sample collection.

### Outcomes

The primary outcome is symptomatic COVID-19, defined by the presence of at least one COVID-19-like symptom as listed in Participants section and confirmed by a positive RT-PCR result for SARS-CoV-2 infection from participants’ NP or nasal swabs.

Secondary outcomes are: (1) symptomatic SARS-CoV-2 infection associated with circulating variants (e.g., Delta, and Omicron); (2) duration of COVID-19 symptoms; (3) hospitalization for COVID-19 within 30 days from enrollment among patients with confirmed COVD-19; (4) need for ICU admission within 30 days from enrollment among patients with confirmed COVD-19; (5) need for invasive mechanical ventilation within 30 days from enrollment among patients with confirmed COVD-19; (6) death from COVID-19 within 90 days from enrollment among patients with confirmed COVD-19; (7) health-related quality of life at 90 days from enrollment among patients with confirmed COVD-19, measured by the three-level version of the EuroQol five-dimensional descriptive system (range, -0.17 [worst] to 1.0 [best]; minimal important difference, 0.03) [7, 8]; (8) persistent COVID-19 symptoms at 180 days from enrollment among patients with confirmed COVD-19; (9) symptomatic SARS-CoV-2 reinfection within 365 days from enrollment among patients with confirmed COVD-19, defined as recurrence of COVID-19-like symptoms confirmed by a positive RT-PCR or antigen test for SARS-CoV-2 more than 90 days after primary infection.

Safety data including adverse events and serious adverse events will be assessed upon inclusion of cases and controls and over the 12-month follow-up for cases, under the standard of care procedures for the city of Toledo’s surveillance program per Brazilian regulatory authority’s requirements.

### Sample size

The sample size was calculated based on formulas published by O’Neill RT [9]. Assuming the recruitment of two controls per case, a vaccine coverage rate of 90% among controls, a sample of 622 cases and 1,244 controls will be required to detect a vaccine effectiveness of 80% with a precision width of 10%. Considering that around 50% of enrolled participants will fulfil criteria for the primary analysis (≥ 2 doses of BNT162b2 or unvaccinated) and increasing the sample size by 20% (due to parameter uncertainties, potential COVID-19 misclassifications, and need for covariate adjustment), the study will need to include 1,500 cases and 3,000 controls.

### Data collection

**Fig 2:**
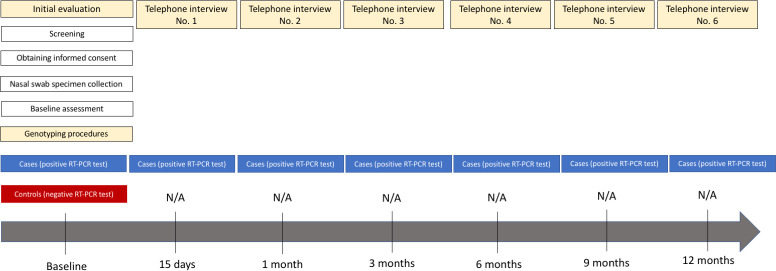
Schedule of enrollment and assessment. N/A. Not appliable; RT-PCR, reverse transcriptase polymerase chain reaction.

The schedule of enrollment and assessment is detailed in Fig 2. Controls are evaluated at a single point in time (baseline). Cases are evaluated at baseline and then followed up for 12 months by means of structured telephone interviews at 15 days, and at 1, 3, 6, 9 and 12 months after inclusion in the study.

Baseline assessment is performed by trained researchers at each participating site at the time an eligible participant with symptoms of COVID-19-like illness seeks care through the public healthcare system. The vaccination status is assessed through structured interviews and adjudicated through review of city medical registry records reviews. Telephone follow-up is conducted via a single central telephone system located in the study coordinating centre (Hospital Moinhos de Vento). Therefore, all participants who are eligible for telephone follow-up (cases), regardless of the site of inclusion, are followed up by researchers trained in good clinical practices and in standardized telephone interview methods. The reference date for scheduling telephone interviews is the date of inclusion in the study. Interviews are considered lost if the participants’ telephone lines are disconnected or non-existent or after 10 attempts at different times on several days within a 5-day time window (5 days after the estimated interview date) for the 15-day interview, and 15-day time window (15 days after the estimated interview date) for the 1-, 3-,6-,9-, and 12-month interviews. Regardless of whether an interview is lost or not, contact is attempted at the subsequent scheduled interviews. This follow-up methodology has been used in previous observational studies [10, 11].

The data are collected using smartphone-, tablet- or computer-based electronic case report forms available on the *Otus* platform (https://site.otus-solutions.com.br/). Access to databases on this platform are granted only by the principal investigator to research team members through a personal, non-transferable username and password. An encrypted digital certificate is used to ensure security in data handling. Platform users (team members) have specific permissions related to their role in the study.

The following procedures have been performed to ensure the quality of the data collected: (1) all researchers have received training in good clinical practices and study procedures, including data collection, before the start of the study; (2) trained personnel at the participating sites are able to contact the study coordinating centre to resolve any questions or problems that they might have; (3) the database is backed up automatically every 12 hours; (4) telephone interviews are recorded and audited to verify data consistency, and the audio files are stored on a server following the same security standards as for the *Otus* platform database; (5) data cleaning is conducted periodically to identify potential inconsistencies in data sets, in which case the researchers are notified for correction; and (6) the coordinating centre have been performing remote monitoring of data quality, revision of detailed reports on screening, inclusion, follow-up, data consistency and completeness on a monthly basis, taking immediate action to resolve any issues, and have been using statistical techniques for identifying fraud throughout the study.

### Collection of biological samples, and diagnostic procedures

A NP or nasal swab sample is collected from all participants for SARS-CoV-2 RT-PCR test and identification of the implicated genetic variant of SARS-CoV-2 at the time of enrollment. This collection is performed by a trained healthcare provider as part of the local policy for surveillance of individuals with suspected or confirmed diagnosis of COVID-19.

All biological materials collected in the study are transported, stored and processed by the Federal University of Paraná (UFPR) according to standardized protocols. NP or nasal swabs are stored in 15 mL falcon tubes containing viral transport medium (VTM) at -80°C, until RT-PCR testing for SARS-CoV-2 and identification of the genetic variants of SARS-CoV-2 are performed.

#### RT-PCR procedures

Total RNA is extracted from the specimens using a viral RNA and DNA kit (MVXA-P096FAST) in an automatic nucleic acid extractor (Extracta 32, Loccus Biotecnologia, Brazil), using magnetic beads. The specimens are then tested to confirm the presence of SARS-CoV-2 genetic material using the BIOMOL OneStep/COVID-19 KIT (IBMP, Curitiba, Brazil) or using the Centers for Disease Control and Prevention 2019-Novel Coronavirus (2019-nCoV) Real-Time RT-PCR Diagnostic Panel (https://www.fda.gov/media/134922/download). This approach has demonstrated high specificity (100%) and sensitivity (88% to 98%) for COVID-19 diagnosis [12, 13].

#### Variant identification

All confirmed positive samples are screened for mutations by RT-PCR to detect SARS-CoV-2 variants as follows: Initially, the multiplex RT-PCR method is performed to detect the Spike Δ69–70 and ORF1a Δ3675–3677 deletions for distinguishing Alpha, Beta/Gamma, wild type/Delta, and Omicron variants (Table 1) [14, 15]. Secondly, the predicted variant is confirmed by two allelic discrimination TaqMan assays (ThermoFisher, USA), which include: K417T, K417N, N501Y, P681R, G339D (Table 2). These assays allow to identify the variants of concern: Alpha (N501Y), Beta (K417N, N501Y), Gamma (K417T, N501Y), Delta (P681R), Omicron BA.1 (N501Y, G339D), and Omicron BA.2 (N501Y, G339D, K417T/N).

**Table 1:** Interpretation of the multiplex RT-PCR for Spike del69/70, Orf1a and N gene.

Spike Del69/70	Orf1a Del3675/3677	N gene	Putative variant
ND	ND	+	Alpha
+	ND	+	Beta/Gamma/BA.2
+	+	+	Wild-type/Delta
ND	+	+	Omicron BA.1

ND: non detected (cycle threshold [CT] value ˃ 40.0); +: positive (CT value ≤ 40.0).

**Table 2:** Identification of SARS-CoV-2 variants by two allelic discrimination assays.

Multiplex assay indication	Two allelic discrimination TaqMan assay	RT-PCR Results	Variant detected
Alpha	K417T	K417T: wt	Alpha
	N501Y	N501Y: mut	
Beta/Gamma/Omicron BA.2	G339D	G339D: wt	Beta
	K417T/N	K417N: mut	
	N501Y	N501Y: mut	
		G339D: wt	Gamma
		K417T: mut	
		N501: mut	
		G339D: mut	Omicron BA.2
		K417T/N: wt	
		N501Y: mut	
Wild/Delta	N501Y	N501Y: wt	Wild-type
	P618R	P681R: wt	
		N501Y: wt	Delta
		P681R: mut	
Omicron BA.1	G339D	G339D: mut	Omicron BA.1
	N501Y	N501Y: mut	

wt: wild-type allele amplification; mut: mutated allele amplification. Any different profile will be classified as “inconclusive”.

Assays are performed using the GoTaq Probe 1-Step RT-qPCR System (Promega) in a 7500 Fast or QuantStudio5 real-time thermocycler (ThermoFisher Scientific Inc., USA), as described by Adamoski et al [16].

### Statistical analysis plan

#### Overall principles

Analysis will start once all data for the last included participant have been obtained, the database has been cleaned and locked, and the study protocol has been submitted for publication. All analyses will be carried out using the R statistical software (R Development Core Team–version to be determined at time of analysis) [17]. Estimate effects will be reported with 95% CIs. A significance level of 0.05 will be used for all statistical comparisons. Secondary analyses and subgroup analyses will not be adjusted for multiple comparisons. They should, therefore, be interpreted as exploratory.

#### Handling missing data

No data imputation will be performed for main exposure (vaccination status) and for the study outcome. Main analysis will consist of observed data, without imputation. For sensitivity analysis we will perform multiple imputation if missing data on core variables is > 5%, under the assumption that the missingness pattern is missing at random conditional on the observed data. Core variables are defined as the covariates to be used in the primary analysis: age, gender, Charlson comorbidity index [18], current smoking status, and body mass index (BMI) category.

#### Participant flow

The flow of participants will be displayed in accordance with the Strengthening the Reporting of Observational Studies in Epidemiology (STROBE) flow diagram (STROBE 2007 statement) [19]. This description will include information about the number of screened for enrollment, reasons for non-participation, number of actually enrolled, and number of analyzed.

#### Baseline characteristics

The baseline characteristics of participants will be presented for the entire study population, test-positive cases, and test-negative controls. Categorical variables will be described as absolute and relative frequencies. Continuous variables will be expressed as mean (standard deviation) for variables with symmetric distribution, and as median (interquartile range) for variables with asymmetric distribution. The symmetry of continuous variables will be checked using graphical analysis of histograms and by the Shapiro-Wilk test.

#### Primary outcome analysis

The primary study analysis compares the vaccine effectiveness for symptomatic SARS-CoV-2 infection of participants vaccinated with two or more doses of BNT162b2 mRNA and participants never vaccinated. Logistic regression adjusted by age, gender, Charlson comorbidity index, BMI, and current smoking status will be used to estimate the odds ratio of vaccination among test positive cases and test negative controls. We also will incorporate in the model the time (number of days) elapsed between the study start and date of RT-PCR sample collection as a proxy of temporal trend. The reference group for vaccination status will be those who had not received a first vaccine dose before the date of sample collection. The estimate of vaccine effectiveness will be provided by 1 –odds ratio x 100 under the assumptions of a test negative design [20]. 95% CIs will be calculated using the Wald method.

#### Sensitivity analyses for the primary outcome

The following sensitivity analyses will be conducted to assess consistency and risk of bias: (1) performing multiple imputation for missing core variables data (see *handling missing data section*); (2) considering only the first enrollment of each individual for the study; (3) excluding as controls those with less than 5 days of symptom onset.

#### Subgroup analyses for the primary outcome

Subgroup analyses, by means of interaction tests, will be conducted to explore potential differences in vaccine effectiveness for symptomatic SARS-CoV-2 infection by age group (12–17, 18–64, and ≥ 65 years), BMI categories, cardiovascular disease (composite of heart failure, ischemic heart disease, cerebrovascular disease, and peripheral arterial disease), and Charlson Comorbidity index ≥ 2.

#### Secondary outcome analyses

The association between the exposition variables with symptomatic SARS-CoV-2 infection caused by circulating variants as well as the association between secondary exposition variables with symptomatic SARS-CoV-2 infection will be assessed using the same statistical model used for the primary outcome.

Crude and adjusted generalized linear models will be used to assess the impact of exposures on duration of COVID-19 symptoms, hospitalisation for COVID-19, need for ICU admission, need for invasive mechanical ventilation, death from COVID-19, health-related quality of life, persistent COVID-19 symptoms, symptomatic SARS-CoV-2 reinfection among participants with a positive RT-PCR result for SARS-CoV-2, and to test associations between length of time after receipt of the second or third dose of BNT162b2 and the risk of symptomatic SARS-CoV-2 infection, according to the probability distribution of each outcome. Quantitative outcomes will be expressed as mean difference. Categorical outcomes will be expressed as hazard ratio or relative risk as appropriate.

### Ethics and dissemination

The study complies with Brazilian National Health Council Resolution no. 466/12 [21]. The study protocol has been approved by the National Commission of Research Ethics (CONEP; CAAE, 50293121.9.0000.5330; opinion number, 4.911,499) and research ethics committee of all participant sites. All participants provide informed consent to participate prior to inclusion in the study. Participants younger than 18 years also provide written informed assent. For persons who lack the cognitive or physical conditions necessary to provide consent, consent to participation is obtained from family proxies.

Results from this study will be disseminated through peer-reviewed publications and conference presentations, addressing primary objective and secondary objectives. Authorship will be defined according to the International Committee of Medical Journal Editors (ICMJE) recommendations [22].

## Study status

The study design and protocol were finalised in October 2021. The first participant was included on November 3, 2021. At study start, approximately 2/3 of the target population was fully vaccinated. Two thousand and thirty-four participants were enrolled up to February 2, 2022. Currently, this study is recruiting participants in 3 referral COVID-19 sites in Toledo. We anticipate that the recruitment will be completed in July 2022.

### Protocol review and amendments

#### Sample size review

According to the original sample size estimation, 2,979 participants (993 cases and 1,986 controls) would be required to detect an odds ratio ≤ 0.70 for the primary outcome analysis, assuming the recruitment of two controls per case, a rate of complete vaccination with the Pfizer-BioNTech BNT162b2 mRNA vaccine of 60% in the control group, a two-tailed alpha of 5%, a power of 80%, and an increased sample size by 30% (due to parameter uncertainties and need for covariate adjustment). In order to achieve adequate power to assess the impact of BNT162b2 on symptomatic SARS-CoV-2 infection associated with the Gamma variant (predominant SARS-COV-2 lineage at the time of study protocol development), considering a rate of 32.5% of genotyping tests with inconclusive results and a prevalence of 90% of the Gamma variant, the original sample size was determined to be 4,500 participants (1,500 cases and 3,000 controls).

In January 2022, the study sample size was revised, given the frequent variation of COVID-19 pandemic factors that could impact sample size estimation (e.g., emergence of Omicron variant, scarcity of new COVID-19 cases due to Gamma variant, increase in vaccine coverage, and vaccine effectiveness variations for distinct SARS-CoV-2 variants). Revision was made based solely on the number of recruited patients, number of cases and proportion of positive cases in the whole sample, without any additional analysis. The original study sample size (4,500 participants) was judged to provide an appropriate power to assess the primary outcome analysis as described in the sample size section.

#### Change in exposure definitions

Given the third vaccine dose of BNT162b2 e became part of standard of care in COVID-19 vaccination in Brazil after study approval, the primary exposure definition was changed from receipt of two doses of BNT162b2 (with the receipt of the second dose 7 days or more before the onset of symptoms of COVID-19-like illness) to receipt of two or more doses of BNT162b2 (with the receipt of the second dose 7 days or more before the onset of symptoms of COVID-19-like illness). We also added the receipt of three doses of Pfizer-BioNTech BNT162b2 mRNA vaccine (with the receipt of the third dose 7 days or more before the onset of symptoms of COVID-19-like illness) as a secondary exposure variable. No data was analysed for this protocol amendment.

## Discussion

The present study has been designed as the first real-world mass vaccination city-wide study with the Pfizer-BioNTech BNT162b2 COVID-19 vaccine in Brazil. Results of this study will allow healthcare professionals, researchers, and policy makers to better understand the real-world effectiveness of BNT162b2 against prevalent variants of concern like Delta and Omicron. In addition, the present study will explore the vaccine effectiveness on relevant, but less explored, outcomes such as persistence of COVID-19 symptoms (i.e., “long COVID-19”) and occurrence symptomatic SARS-CoV-2 re-infections.

The test-negative design was chosen due to its feasibility and reliability of findings in a real-world mass vaccination context. Test-negative design studies have been extensively used to estimate vaccine effectiveness against symptomatic respiratory infections [23], including COVID-19 [24–26], while controlling for potential biases such as healthcare seeking behaviour and misclassification of COVID-19 status. Strengths of our study include the prospective ascertainment of infection status to guarantee the inclusion of case and controls with similar disease manifestation—preventing sample bias; the use of adjudication procedures to avoid recall bias of exposure factors (i.e., vaccination status); the genotyping procedures of all COVID-19 cases—allowing the assessment of vaccine effectiveness against specific SARS-CoV-2 variants of concern; and the prospective follow-up of cases to allow the evaluation of vaccine impact on COVID-19-related complications.

Our study has some limitations. First the present study design is susceptible to confounding due to differences between vaccinated and unvaccinated persons that may influence the occurrence of COVID-19. In order to minimize this potential bias, relevant risk factors for COVID-19 (both person- and time-related variables) will be used for covariate adjustment in the primary analyses. Moreover, sensitivity analyses will be conducted to assess consistency of results. Second, the sample size calculation may be inaccurate, given premises used to calculate the number of participants are highly variable based on the prevalence of COVID-19 in the study period. Third, misclassifications of cases and controls are possible, given the accuracy of RT-PCR for COVID-19 is not perfect. Nevertheless, the study protocol includes actions aimed to optimize diagnosis accuracy (e.g., exclusion of individuals at increased risk for false negative tests, use of validated RT-PCR test procedures with high sensitivity and specificity, and sensitivity analyses to assess consistency of findings under distinct test accuracy scenarios). Fourth, modifications in the standard of care regarding COVID-19 vaccination demanded changes in the definition of exposure variables to reflect the real-world practices while keeping the study procedures feasible.

Evidence regarding effectiveness of BNT162b2 in Brazil and in the southern hemisphere is limited and this study will help fill this important epidemiological gap. These data can broaden the evidence-base from clinical trials help guide effective COVID-19 vaccination policies.

## Supporting information

S1 File (PDF)
