# Supplementary material for: BNT162b2 against COVID-19 in Brazil using a test-negative design: Study protocol and statistical analysis plan
Source: PLoS One. 2022 Oct 20;17(10):e0276384. doi: 10.1371/journal.pone.0276384 (PMC9584383; doi:10.1371/journal.pone.0276384)
Supplement: S1 File — (PDF) [file pone.0276384.s001.pdf]

RESEARCH ETHICS COMMITTEE OPINION

DATA ON THE RESEARCH PROJECT

Research Project Title: Impact of the Pfizer-BioNTech BNT162b2 mRNA vaccine on the prevention of symptomatic infection, hospitalization, and death from SARS-CoV-2 in a Southern Brazilian city: a real-world study.

Investigator: Regis Goulart Rosa

Research Track: Research projects involving genetically modified organisms (GMOs), embryonic stem cells and organisms, that represent a high collective risk, including organisms related thereto, regarding: experimentation, construction, cultivation, manipulation, transport, transfer, import, export, storage, environmental release, and disposal;
Research coordinated and/or sponsored from outside Brazil, except those co-sponsored by the Brazilian Government;

Version: 2

Certificate of Submission for Ethical Approval: 50293121.9.0000.5330

Proposing Institution: Hospital Moinhos de Vento - HMV

Lead Sponsor: Hospital Moinhos de Vento - HMV

DATA ON THE ETHICS COMMITTEE OPINION

Opinion Number: 4,911,499

Project Overview:

The information listed in the fields “Project Overview”, “Study Objective”, and “Assessment of Risks and Benefits” were obtained from the file “PB_INFORMAÇÕES_BÁSICAS_DO_PROJETO_1798851.pdf”, dated August 13, 2021.

INTRODUCTION

COVID-19, the infection caused by the novel coronavirus SARS-CoV-2, has already affected millions of people worldwide. In Brazil, over 19 million cases and 500,000 deaths from COVID-19 have already been reported. Currently, less than 15% of the Brazilian population is fully immunized. As a result, mass vaccination campaigns using recently approved SARS-CoV-2 vaccines have become a health priority. The Pfizer-BioNTech mRNA vaccine, BNT162b2, was recently approved for use in individuals aged 12 years or over by the Brazilian National Health Surveillance Agency (ANVISA). In a recent randomized controlled trial (RCT) of 43,448 participants aged

16 years or older, the 2-dose Pfizer-BioNTech BNT162b2 mRNA vaccine regimen demonstrated 95% efficacy (95% confidence interval [95%CI], 90.3% to 97.6%) against laboratory-confirmed SARS-CoV-2 infection. Vaccine-related adverse events (mainly pain at the injection site, fatigue, and headache) were reported in 21% of participants, being classified as mild to moderate in most cases. In a subsequent RCT of 2,260 adolescents aged 12 to 16 years, the 2-dose Pfizer-BioNTech BNT162b2 mRNA vaccine regimen demonstrated 100% efficacy (95%CI, 75.3% to 100%) against laboratory-confirmed SARS-CoV-2 infection. The adverse effect profile was considered safe by the authors, with a predominance of pain at the injection site, fatigue, and headache, again ranging from mild to moderate. However, there is still little evidence of the efficacy of the Pfizer-BioNTech BNT162b2 mRNA vaccine in contexts of high prevalence of SARS-CoV-2 variants of concern, as is the case of the Gamma (P.1) variant (currently the most prevalent strain in Brazil overall and in the state of Paraná), of its long-term protection and risks, and of its impact on COVID-19 disease severity and post-COVID conditions. Although RCTs are the gold standard for assessing the efficacy and safety of COVID-19 vaccines, real-world evidence, particularly from observational studies of vaccination, are increasingly needed to validate the findings of RCTs in a broad and diverse range of individuals and health practices, including across different treatment patterns, dosages, intervals between doses, adherence, and in settings of high prevalence of SARS-CoV-2 variants of concern. In this scenario, real-world vaccination studies have been conducted to assess the efficacy and safety of recently approved SARS-CoV-2 vaccines in different health contexts,[7-10] contributing to the construction of a relevant evidence base regarding the efficacy and safety of COVID-19 vaccines – a global priority. In Brazil, real-world research initiatives have been implemented to assess the effectiveness of recently approved COVID-19 vaccines in the context of mass vaccination campaigns. In Serrana, a town of just over 45,000 in the state of São Paulo, symptomatic cases of COVID-19 and COVID-related hospital admissions declined 80% and 86%, respectively, after mass vaccination with the Sinovac-CoronaVac vaccine. A similar experiment is underway in another city in São Paulo. The city of Botucatu hopes to immunize most of its 148,000 inhabitants with the Oxford-AstraZeneca vaccine. Although still incipient, mass vaccination campaigns with nested observational studies are expected to become more frequent in Brazil as part of strategies to contain the spread of COVID-19 and to assess the real-world effectiveness of recently approved vaccines in a setting of high prevalence of SARS-CoV-2 variants of concern.

HYPOTHESIS

The investigators hypothesize that, compared to no vaccination, full vaccination with the Pfizer-BioNTech BNT162b2 mRNA vaccine in the real-world context of the city of Toledo, Paraná, will be associated with a reduction in the odds of symptomatic SARS-CoV-2 infection.

METHODS

This is an observational study with a hybrid design, namely, a case-control study with a prospective cohort for follow-up of confirmed cases. This design is commonly employed in evaluating the real-world effectiveness of vaccination programs. In this design, cases will be defined as individuals who seek care at public health facilities with signs, symptoms, or an overall clinical picture suspicious for COVID-19 and who test positive for SARS-CoV-2. Controls will be defined as individuals who seek care at public health facilities with signs, symptoms, or a clinical picture suspicious for COVID-19, but test negative for SARS-CoV-2. Cases will be followed up for 12 months through structured, centralized telephone interviews. Thus, while the case-control design will allow us to identify the impact of vaccination on the prevention of symptomatic COVID-19 infection, the cohort study will allow us to assess the impact of vaccination on the severity of
confirmed cases.

INCLUSION CRITERIA

Inclusion criteria for cases and controls:

Age 12 years or older;

Live in the city of Toledo, Paraná;

Seek care at a public health facility with symptoms or a clinical picture indicative of COVID-19*;

Nasal swab sample collected for SARS-CoV-2 PCR as part of the standard of care.

EXCLUSION CRITERIA

Exclusion criteria for cases and controls:

Treatment with remdesivir within the last 30 days;

Treatment with convalescent plasma within the last 90 days;

Treatment with any monoclonal antibodies against COVID-19 (e.g., casirivimab/imdevimab, bamlanivimab, etesivumab) within the last 90 days;

Absence of consent for study participation.

Research Objective:

PRIMARY OBJECTIVE

This study aims to evaluate the real-world effectiveness of the Pfizer-BioNTech BNT162b2 mRNA vaccine in the city of Toledo, Paraná.

Risk and Benefit Assessment:

RISKS

The risks to the participant include those inherent to the collection of biological material and discomfort associated with answering health-related questions. In these cases, the participant may choose not to respond. Collection of respiratory secretions carries risks inherent to nasal swab sampling, such as discomfort, local pain, slight nosebleed, vomiting, or cough due to the swab procedure. For the purposes of this study, an additional nasal swab will be collected to that already routinely obtained by the health facility, for a total of two nasal swabs: one specimen for the diagnosis of COVID-19 (standard of care) and one additional sample for SARS-CoV-2 genotyping (study procedure). The procedure will be performed by duly trained health personnel capable of assessing the patient's condition and stopping the procedure if necessary. All sample collections will be carried out using disposable, single-use material, and are therefore devoid of any risk of contamination. Any complications arising from the study will be evaluated and monitored health personnel from the participating centers and the proponent central center. If any injury or harm to health is proven to have occurred as a result of participation in the study, comprehensive care will be made available for as long as necessary, free of charge.

BENEFITS

Benefit to the participant is indirect. The results of this study will translate into knowledge that can clarify or improve future approaches to vaccination.

Notes and Remarks on the Study:

Responses to CONEP Opinion No. 4,895,402, issued August 9, 2021

Observational study to evaluate the real-world effectiveness of the Pfizer-BioNTech BNT162b2 mRNA vaccine in the city of Toledo, Paraná. Case-control study with a prospective cohort for identified cases. Participants aged 12 or over who meet eligibility criteria and who seek care at public health facilities in the city of Toledo, Paraná, with symptoms suggestive of COVID-19 will be included. Participants will be classified as cases (RT-PCR test positive for SARS-CoV-2) or controls (RT-PCR test negative for SARS-CoV-2).

The primary exposure variable will be full vaccination (2 doses) with the Pfizer-BioNTech BNT162b2 COVID-19 mRNA vaccine versus never-vaccinated for COVID-19 status.

For participants classified as cases, data will be collected at seven time points over 12 months of follow-up; for participants classified as controls, data will be collected at the time of initial assessment. Data will be collected on: sociodemographic parameters; comorbidities; vaccination status; adverse reactions to the vaccine; type of presenting encounter; presenting symptoms; previous history of diagnosis of COVID-19; result of SARS-CoV-2 PCR from nasal swab; result of SARS-CoV-2 genotyping; vital status; duration of symptoms; hospitalizations; ICU admission; need for mechanical ventilation; SARS-CoV-2 reinfection; and quality of life (EQ5D-3L). Data from the initial assessment will be those routinely collected by the epidemiological surveillance department of the Toledo Municipal Department of Health from individuals who undergo SARS-CoV-2 PCR due to clinical suspicion of COVID-19. Outcome data will be extracted from SARS-CoV-2 PCR and genotyping, as well as from telephone interviews.

The study is expected to enroll 4,500 participants (1,500 cases and 3,000

controls). Sponsor: Hospital Moinhos de Vento

Co-participating institutions: Toledo Municipal Department of Health (screening for eligibility, obtaining informed consent, enrolling participants in the study at municipal referral units for care/diagnosis of COVID-19) and Universidade Federal do Paraná (identification of SARS-CoV-2 variants).

Funding: Pfizer Brasil

Expected study start date: August 30, 2021
Expected study completion date: July 31, 2023

Remarks on Mandatory Filings:
See "Conclusions or Outstanding Issues and List of Inadequacies".

Conclusions or Outstanding Issues and List of Inadequacies:
Analysis of responses to Opinion No. 4,895,402, issued by Conep on August 9, 2021:

1. Regarding the research project:

1.1. The document "Protocol_Pfizer_BioNTech_BNT162b2_27_Julho.pdf" reads "Funding: Pfizer Brasil is the source of funding for the present study". However, the documents "PB_INFORMAÇÕES_BÁSICAS_DO_PROJETO_1798851.pdf", dated July 30, 2021, and "Folha_de_Rosto_final.pdf" state "Funding [...] Own funding". Clarification is hereby requested regarding the actual sponsor of the study, adequacy of project registration in Plataforma Brasil, and submission of a new Cover Sheet (National Health Council Operating Standard No. 001/2013, item 3.3.e). RESPONSE: The study sponsor is Hospital Moinhos de Vento (HMV). Pfizer Brasil will provide financial support for the present study. This information has been updated in the project cover sheet as well as in version 2 of the protocol (documents: " Protocolo_Pfizer_BioNTech_BNT162b2_12_Ago_2021_V2_marcado and Protocolo_Pfizer_BioNTech_BNT162b2_12_Ago_2021_V2_clean", .pdf and .docx).
ASSESSMENT: OUTSTANDING ISSUE ADDRESSED.

1.2. Page 20 of 40 of the document "Protocol_Pfizer_BioNTech_BNT162b2_27_Julho.pdf" reads "One swab will be sent to the Paraná State Central Laboratory (LACEM-PR) for SARS-CoV-2 PCR testing, according to the standard of care at Toledo-PR. The other swab will be stored at the Toledo Campus of UFPR at -70°C, until final transport to Universidade Federal do Paraná - Curitiba for identification of SARS-CoV-2 genetic variants".

Since there will be storage of biological samples, all aspects related to the establishment of a biorepository during the conduct of the study must be followed as explained in National Health Council Resolution No. 441/11 and Ministry of Health Ordinance No. 2201/11, even if the storage of biological material is temporary and no future use is anticipated. It is hereby requested that the aforementioned regulations be reviewed, and the following submitted:

1.2.1. Rationale as to the need and opportunity for future utilization of biological specimens stored as part of the proposed study (Item 2.I of National Health Council Resolution No. 441/11), if relevant. RESPONSE: According to the Memorandum of Agreement on Establishment of a Biorepository (document: Termo_Acordo_Biorrepositorio.pdf), such a rationale does not apply to the proposed study, as there is no intention for future use of the stored biological specimens. Specimens will only be used for the purposes described in the protocol.
ASSESSMENT: OUTSTANDING ISSUE ADDRESSED.

1.2.2. Statement that any new research to be carried out on material stored in the biorepository will be submitted for approval by the institutional Research Ethics Committee and, when applicable, by the National Research Ethics Committee (CONEP) (Item 2.III, National Health Council Resolution 441/11), if relevant.
RESPONSE: According to the Memorandum of Agreement on Constitution of a Biorepository (document: Termo_Acordo_Biorrepositorio.pdf), such a statement does not apply to the proposed study, as there is no intention for future use of the stored biological specimens. Specimens will only be used for the purposes described in the protocol.
ASSESSMENT: OUTSTANDING ISSUE ADDRESSED.

1.2.3. Regulation of laboratories involved in the storage of biological material: operational and infrastructure details, as well as conditions for storage of said material, which may be contained in the detailed research project or in the form of a statement. It bears stressing that the storage period for human biological material in a biorepository must be in accordance with the corresponding research timetable, and may be authorized for up to 10 years.

RESPONSE: Operational and infrastructure details, as well as the conditions for storage of the material, are described in the Establishment of a Biorepository item of the protocol (version 2). For a clearer understanding of the changes made, version 2 of the protocol has been uploaded with tracked changes (documents: " Protocolo_Pfizer_BioNTech_BNT162b2_12_Ago_2021_V2_marcado and Protocolo_Pfizer_BioNTech_BNT162b2_12_Ago_2021_V2_clean", .pdf and .docx). ASSESSMENT: OUTSTANDING ISSUE ADDRESSED.

1.2.4. Memorandum of Agreement for the establishment of a biorepository of human biological material involving more than one institution, covering means for operationalization, sharing, use, and disposal after use of human biological material stored in the biorepository, including the possibility of future dissolution of the partnership and the consequent sharing and disposition of stored data and materials, signed by the investigators in charge at each participating institution and their institutional officers ("Guidance handbook: frequent outstanding issues in clinical research", version 1.0, 2015). RESPONSE: We have uploaded the Memorandum of Agreement as an attachment through Plataforma Brasil (document: Termo_Acordo_Biorrepositorio.pdf).
ASSESSMENT: OUTSTANDING ISSUE ADDRESSED.

1.2.5. The document "PB_INFORMAÇÕES_BÁSICAS_DO_PROJETO_1798851.pdf", dated July 30, 2021, states that samples will not be retained for storage in a "bank". The term "bank" is misinterpreted as "biobank", when it actually applies to both biobanks and biorepositories. Thus, whenever there is any collection and/or storage of biological material for research purposes, this field in Plataforma Brasil must be marked "YES". It is hereby requested that the ICF be amended accordingly.
RESPONSE: Amended accordingly.
ASSESSMENT: OUTSTANDING
ISSUE ADDRESSED.

2. Regarding the documents "TCLE_Participante_RL_18anos_V1_28JUL2021.pdf" and "TCLE_RL_12_17anos_V1_28_JUL_2021.pdf", uploaded on July 30, 2021:

2.1. Page 3 of 5 reads: "However, if [participants] must travel specifically for a research procedure, the investigators will ensure reimbursement of their transportation expenses". Research participants and their chaperones must be ensured reimbursement of any expenses arising from participation in the trial whenever their presence is required for study visits, tests, or examinations. Therefore, it is hereby requested that the text be rewritten to state, in clear and affirmative wording, that trial participants and their chaperones will be reimbursed any expenses incurred as a result of their participation, including, but not limited to, transportation and food (National Health Council Resolution 466/12, items II.21 and IV.3.g).
RESPONSE: Changes have been made, generating version 2 of the corresponding forms. For a clearer understanding of the changes made, version 2 of the ICFs have been uploaded with tracked changes (documents: T C L E _ P a r t i c i p a n t e _ R L _ 1 8 a n o s _ V 2 _ 1 2 _ A G O _ 2 0 2 1 _ m a r c a d o , T C L E _ P a r t i c i p a n t e _ R L _ 1 8 a n o s _ V 2 _ 1 2 _ A G O _ 2 0 2 1 _ c l e a n , TCLE_RL_12-17anos_V2_12_AGO_2021_marcado and
TCLE_RL_12-17anos_V2_12_AGO_2021_clean em .pdf e .docx)
ASSESSMENT: OUTSTANDING ISSUE ADDRESSED.

2.2. It is hereby requested that the ICF be amended to include an explanation regarding the right to seek compensation in case of any harm arising from participation in the study (National Health Council Resolution No. 466/12, item IV.3.h).
RESPONSE: Changes have been made, generating version 2 of the corresponding forms. For a clearer understanding of the changes made, version 2 of the ICFs and Informed Assent Form have been uploaded with tracked changes (documents:
T C L E _ P a r t i c i p a n t e _ R L _ 1 8 a n o s _ V 2 _ 1 2 _ A G O _ 2 0 2 1 _ m a r c a d o , T C L E _ P a r t i c i p a n t e _ R L _ 1 8 a n o s _ V 2 _ 1 2 _ A G O _ 2 0 2 1 _ c l e a n , TCLE_RL_12-17anos_V2_12_AGO_2021_marcado and
TCLE_RL_12-17anos_V2_12_AGO_2021_clean, .pdf and .docx)
TALE_12anos_V2_12_AGO_2021_marcado and
TALE_12anos_V2_12_AGO_2021_clean, .pdf and .docx).
ASSESSMENT: OUTSTANDING ISSUE ADDRESSED.

2.3. Please include contact information for Conep in the ICF (Comissão Nacional de Ética em Pesquisa – Conep SRTV 701, Via W 5 Norte, lote D - Edifício PO 700, 3º andar – Asa Norte CEP:

70719-040, Brasília/DF).

RESPONSE: Changes have been made, generating version 2 of the corresponding forms. For a clearer understanding of the changes made, version 2 of the ICFs have been uploaded with tracked changes (documents: T C L E _ P a r t i c i p a n t e _ R L _ 1 8 a n o s _ V 2 _ 1 2 _ A G O _ 2 0 2 1 _ m a r c a d o , T C L E _ P a r t i c i p a n t e _ R L _ 1 8 a n o s _ V 2 _ 1 2 _ A G O _ 2 0 2 1 _ c l e a n , TCLE_RL_12-17anos_V2_12_AGO_2021_marcado and TCLE_RL_12-17anos_V2_12_AGO_2021_clean em .pdf e .docx)

ASSESSMENT: OUTSTANDING ISSUE ADDRESSED.

Final Considerations at the Discretion of the Committee:

In view of the foregoing, the National Research Ethics Committee (Conep), in accordance with the mandates set forth in Brazilian National Health Council Resolution No. 466/2012 and Operational Standard No. 001/2013, moves to approve the proposed research project.

Opinion Status: Protocol approved.

This opinion was reached on the basis of the documents listed below:

Document Type:	File	Posted	Auth or	Status
Basic Project Information	PB_INFORMAÇÕES_BÁSICAS_DO_PROJETO_1798851.pdf	8/13/2021 13:48:51		Accepted
Cover Sheet	Folha_de_rosto_assinada.pdf	8/13/2021 13:47:47	Regis Goulart Rosa	Accepted
Other	Carta_Resposta_Pendencias_CONEP_PC_4895402_assinadopdf.pdf	8/13/2021 13:46:46	Regis Goulart Rosa	Accepted
Other	Carta_Resposta_Pendencias_CONEP_PC_4895402.docx	8/13/2021 13:40:51	Regis Goulart Rosa	Accepted
Detailed Project / Investigator Brochure	Protocolo_Pfizer_BioNTech_BNT162b2_12_Ago_2021_V2_marcado.pdf	8/13/2021 13:40:19	Regis Goulart Rosa	Accepted
Detailed Project / Investigator Brochure	Protocolo_Pfizer_BioNTech_BNT162b2_12_Ago_2021_V2_marcado.docx	8/13/2021 13:39:59	Regis Goulart Rosa	Accepted
Detailed Project / Investigator Brochure	_Protocolo_Pfizer_BioNTech_BNT162b2_12_Ago_2021_V2_clean.pdf	8/13/2021 13:39:42	Regis Goulart Rosa	Accepted
Detailed Project / Investigator Brochure	_Protocolo_Pfizer_BioNTech_BNT162b2_12_Ago_2021_V2_clean.docx	8/13/2021 13:39:24	Regis Goulart Rosa	Accepted

Informed Consent Form / Assent Form / Rationale for Absence Thereof	TCLE_RL_12_17anos_V2_12_AGO_2021_marcado.pdf	8/13/2021 13:38:49	Regis Goulart Rosa	Accepted
Informed Consent Form / Assent Form / Rationale for Absence Thereof	TCLE_RL_12_17anos_V2_12_AGO_2021_marcado.docx	8/13/2021 13:38:32	Regis Goulart Rosa	Accepted
Informed Consent Form / Assent Form / Rationale for Absence Thereof	TCLE_RL_12_17anos_V2_12_AGO_2021_clean.pdf	8/13/2021 13:38:00	Regis Goulart Rosa	Accepted
Informed Consent Form / Assent Form / Rationale for Absence Thereof	TCLE_RL_12_17anos_V2_12_AGO_2021_clean.docx	8/13/2021 13:37:44	Regis Goulart Rosa	Accepted
Informed Consent Form / Assent Form / Rationale for Absence Thereof	TCLE_Participante_RL_18anos_V2_12_AGO_2021_marcado.pdf	8/13/2021 13:36:21	Regis Goulart Rosa	Accepted
Informed Consent Form / Assent Form / Rationale for Absence Thereof	TCLE_Participante_RL_18anos_V2_12_AGO_2021_marcado.docx	8/13/2021 13:35:55	Regis Goulart Rosa	Accepted
Informed Consent Form / Assent Form / Rationale for Absence Thereof	TCLE_Participante_RL_18anos_V2_12_AGO_2021_clean.pdf	8/13/2021 13:35:34	Regis Goulart Rosa	Accepted
Informed Consent Form / Assent Form / Rationale for Absence Thereof	TCLE_Participante_RL_18anos_V2_12_AGO_2021_clean.docx	8/13/2021 13:34:57	Regis Goulart Rosa	Accepted

Informed Consent Form / Assent Form / Rationale for Absence Thereof	TALE_12anos_V2_12_AGO_2021_marcado.pdf	8/13/2021 13:34:32	Regis Goulart Rosa	Accepted
Informed Consent Form / Assent Form / Rationale for Absence Thereof	TALE_12anos_V2_12_AGO_2021_marcado.docx	8/13/2021 13:34:14	Regis Goulart Rosa	Accepted
Informed Consent Form / Assent Form / Rationale for Absence Thereof	TALE_12anos_V2_12_AGO_2021_clean.pdf	8/13/2021 13:33:49	Regis Goulart Rosa	Accepted
Informed Consent Form / Assent Form / Rationale for	TALE_12anos_V2_12_AGO_2021_clean.docx	8/13/2021 13:33:11	Regis Goulart Rosa	Accepted

Absence Thereof	TALE_12anos_V2_12_AGO_2021_clean.docx	8/13/2021 13:33:11	Regis Goulart Rosa	Accepted
Statement on Handling of Biological Material / Biorepository / Biobank	Termo_Acordo_Biorrepositorio.docx	8/13/2021 13:32:51	Regis Goulart Rosa	Accepted
Statement of Statement on Handling of Biological Material / Biorepository / Biobank	Termo_Acordo_Biorrepositorio.pdf	8/13/2021 13:31:51	Regis Goulart Rosa	Accepted
Other	HMV_Carta_de_Submissao_29_Julho.pdf	7/30/2021 14:32:13	Regis Goulart Rosa	Accepted
Detailed Project / Investigator Brochure	Protocolo_Pfizer_BioNTech_BNT162b2_27_Julho.docx	7/30/2021 14:31:25	Regis Goulart Rosa	Accepted
Detailed Project / Investigator Brochure	Protocolo_Pfizer_BioNTech_BNT162b2_27_Julho.pdf	7/30/2021 14:30:22	Regis Goulart Rosa	Accepted
Other	Declaracao_de_compromisso_Equipe_de_pesquisa.pdf	7/30/2021 14:28:43	Regis Goulart Rosa	Accepted
Other	Concordancia_Realizacao_Servicos_NFN.pdf	7/30/2021 14:27:34	Regis Goulart Rosa	Accepted
Other	HMV_Formulario_de_Autorizacao_para_Submissao_de_Projeto_de_Pesquisa_ao_CEP.pdf	7/30/2021 14:09:54	Regis Goulart Rosa	Accepted
Other	Parecer_da_Comissao_Cientifica_ID275_PDF.pdf	7/30/2021 14:09:16	Regis Goulart Rosa	Accepted
Other	Ad_Referendum_SMS_Toledo.pdf	7/30/2021 14:08:42	Regis Goulart Rosa	Accepted
Other	Fichas_de_Coleta_de_Dados.pdf	7/30/2021 14:08:24	Regis Goulart Rosa	Accepted
Informed Consent Form / Assent Form / Rationale for Absence Thereof	TALE_12anosV1_28JUL2021.pdf	7/30/2021 14:07:55	Regis Goulart Rosa	Accepted
Informed Consent Form / Assent Form / Rationale for Absence Thereof	TCLE_Participante_RL_18anos_V1_28JUL2021.pdf	7/30/2021 14:07:44	Regis Goulart Rosa	Accepted

Informed Consent Form / Assent Form / Rationale for Absence Thereof	TCLE_RL_12_17anos_V1_28_JUL_2021.pdf	7/30/2021 14:07:30	Regis Goulart Rosa	Accepted
Other	Concordancia_Servicos_CampusToledoUFPR.pdf	7/30/2021 14:04:46	Regis Goulart Rosa	Accepted

Other	Coparticipacao_SMS_Toledo.pdf	7/30/2021 14:00:22	Regis Goulart Rosa	Accepted
Other	Concordancia_de_coparticipacao_UFPR.pdf	7/30/2021 14:00:00	Regis Goulart Rosa	Accepted
Statement of Site and Infrastructure	Declaracao_infraestrutura_SMS_Toledo.pdf	7/30/2021 13:59:30	Regis Goulart Rosa	Accepted
Statement of Site and Infrastructure	Declaracao_infraestrutura_UFPR.pdf	7/30/2021 13:59:13	Regis Goulart Rosa	Accepted
Other	HMV_Termo_de_compromisso_do_pesquisador_responsavel_e_da_instituicao.pdf	7/30/2021 13:58:16	Regis Goulart Rosa	Accepted
Other	HMV_Termo_de_compromisso_do_pesquisador_responsavel_e_da_instituicao.doc	7/30/2021 13:58:00	Regis Goulart Rosa	Accepted
Statement of Statement on Handling of Biological Material / Biorepository / Biobank	HMV_Termo_de_compromisso_de_utilizacao_do_material_biologico.pdf	7/30/2021 13:57:20	Regis Goulart Rosa	Accepted
Statement of Statement on Handling of Biological Material / Biorepository / Biobank	HMV_Termo_de_compromisso_de_utilizacao_do_material_biologico.doc	7/30/2021 13:57:02	Regis Goulart Rosa	Accepted
Statement of Site and Infrastructure	HMV_Declaracao_de_Infraestrutura_e_instalacoes.docx	7/30/2021 13:56:21	Regis Goulart Rosa	Accepted
Statement of Site and Infrastructure	HMV_Declaracao_de_Infraestrutura_e_instalacoes.pdf	7/30/2021 13:55:52	Regis Goulart Rosa	Accepted
Other	HMV_Plano_de_recrutamento_dos_sujeitos_circunstancias_de_obtencao_do_TCLE_e_quem_ira_obte_lo.pdf	7/30/2021 13:55:06	Regis Goulart Rosa	Accepted
Other	HMV_Plano_de_recrutamento_dos_sujeitos_circunstancias_de_obtencao_do_TCLE_e_quem_ira_obte_lo.doc	7/30/2021 13:54:52	Regis Goulart Rosa	Accepted
Other	HMV_Declaracao_de_confidencialidade.pdf	7/30/2021 13:48:56	Regis Goulart Rosa	Accepted
Other	HMV_Declaracao_de_confidencialidade.doc	7/30/2021 13:48:29	Regis Goulart Rosa	Accepted

Opinion Status:
Accepted

BRASILIA, August 17, 2021

Signed By:
Jorge Alves de Almeida Venancio
(Coordinator)
